# Complete remission of Hodgkin’s lymphoma in a pediatric patient with TTN gene mutation treated with brentuximab vedotin combined chemotherapy without anthracyclines: A case report

**DOI:** 10.3389/fonc.2022.1006166

**Published:** 2022-10-17

**Authors:** Ying Li, Ligang Liu, Hao Sun, Nan Li, Shuang Huang, Alexander Olinger, Xiaolin Xu, Xiaoling Wang, Yanlong Duan

**Affiliations:** ^1^ Department of Pharmacy, Beijing Children’s Hospital, Capital Medical University, National Center for Children’s Health, Beijing, China; ^2^ College of Pharmacy, The Ohio State University, Columbus, OH, United States; ^3^ Department of Pharmacy, Dalian Women and Children’s Medical Center (Group), Dalian, China; ^4^ Medical Oncology Department, Pediatric Oncology Center, Beijing Children’s Hospital, National Center for Children’s Health, Beijing Key Laboratory of Pediatric Hematology Oncology, Key Laboratory of Major Diseases in Children, Ministry of Education, Capital Medical University, Beijing, China; ^5^ Department of Pharmacy, Nebraska Medicine, Omaha, NE, United States

**Keywords:** TTN gene mutation, cardiotoxicity, brentuximab vedotin, Hodgkin’s lymphoma (HL), TTN

## Abstract

**Introduction:**

There is no guideline for the treatment of Hodgkin’s lymphoma (HL) in pediatric patients with titin (TTN) gene mutation and heart failure. We explored the feasibility of using brentuximab vedotin (BV) plus chemotherapy without anthracyclines to treat one pediatric HL patient with TTN mutation.

**Case presentation:**

A 5-year and 7-month male patient was admitted to the hospital due to high fever and shortness of breath. He was diagnosed with stage IV IVB high-risk Hodgkin’s lymphoma (lymphocyte-depleted type) at admission. Echocardiography showed that the left ventricular ejection fraction (LVEF) was 27%. The gene sequencing revealed a pathogenic variant in the TTN gene. Due to the risk of cardiotoxicity of anthracycline, he received 6 cycles of chemotherapy (no anthracyclines), 4 cycles of them plus BV with dosing 1.8 mg/kg, q3w. The tumor was reduced by 77% after 2 cycles of BV and 4 cycles of chemotherapy. At the end of 4 cycles of BV and six courses of chemotherapy, with complete remission achieved, the tumor was reduced by 85%. After 11 months of follow-up, the patient was still in complete remission with no adverse events reported, and his LVEF improved to 62%.

**Conclusion:**

The combination of BV with chemotherapy is effective and well-tolerated for pediatric HL patients with TTN gene mutation.

## Introduction

Hodgkin’s lymphoma (HL) is a malignant tumor with a good prognosis (1). Anthracyclines, vinca alkaloids, glucocorticoids, and alkylating agents are typical cornerstones of therapy for HL (2). However, for patients with titin (TTN) gene mutation, some previous studies have reported that they are prone to secondary cardiomyopathy after receiving anthracyclines containing chemotherapy regimens (3) and even sudden cardiac death (4). Brentuximab vedotin (BV) is an antibody–drug conjugate, which combines a CD30 monoclonal antibody with the potent antimicrotubular agent, monomethyl auristatin E (MMAE) (5), and it has shown a significant clinical benefit and tolerated toxicity for HL patients (5, 6).

So far, there is no study or case report that focuses on the treatment of HL in patients with TTN gene mutation and impaired heart function. Therefore, this case is aimed to explore the efficacy and safety of using BV to displace anthracycline in the initial treatment plan in one patient with TTN mutation. This case report was prepared strictly following the CARE Guidelines (7).

## Case presentation

A 5-year and 7-month-old boy presented to the oncology clinic with a history of bilateral cervical lymph node enlargement for 3 years, intermittent fever for 1 month, and tachypnea for 15 days. Pollen pini was used to manage the symptoms in the past 3 years. However, intermittent fever occurred about every 15 days during the treatment, which was relieved by physical cooling combined with oral antipyretic medications. No significant weight reduction or apparent changes in cervical lymph nodes were observed during the treatment. Two months ago, the patient stopped taking Pollen pini due to COVID-19 pandemic restriction. His parents and two sisters are healthy, with no known significant diseases.

On initial presentation, he was 116 cm and 19.5 kg, his body surface area (BSA) was 0.765 m^2^, and his BMI was 14.49 kg/m^2^. On physical examination, several soft, non-tender swollen lymph nodes could be touched in the left neck with a good range of motion. The largest was about 2 × 3 cm. He looked pale, with an anemic appearance. The heart rhythm was regular, and no murmurs were heard. His heart rate was 102 bpm when he arrived at the ward.

His complete blood counts showed white blood cells 5.56 × 10^9^/l, Hb 82 g/l, and platelet count 172 × 10^9^/l. His abdominal CT showed enlarged liver, spleen, and kidneys and multiple enlarged lymph nodes behind the diaphragmatic angle, retroperitoneum, and intra-abdominal cavity. Echocardiography indicated a left ventricular ejection fraction (LVEF) of 27%, moderate tricuspid regurgitation, and low to mild mitral regurgitation. A 24-h Holter monitor showed an average heart rate of 136 bpm and a Childhood Hodgkin International Prognostic Score (CHIPS) of 2.

**Figure 1:**
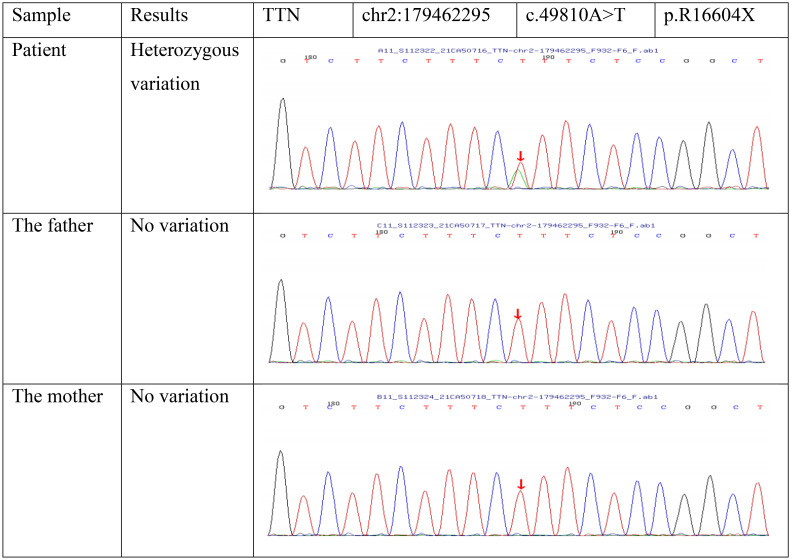
Results of family analysis of TTN gene variation.

Lymph node pathological biopsy results showed classic Hodgkin lymphoma (lymphocyte depleted cHL, LDCHL). Immunohistochemical staining (IHC) demonstrated large cells CD3(-), CD20(-), CD30**(+)**, ALK (-), CD68 (-), MUM1(+) Ki-67(+), LCA (-), BOB1(-), OCT2 (weak +), PAX-5 (weak +), CD4(-), LMP (+), PD- 1(-), CD15(-), and BcL-2(NS). *In situ* hybridization results showed EBER (+). Whole exome sequencing (WES) by next-generation sequencing for this patient and his parents identified that only the patient has a spontaneous likely pathogenic variant in the TTN gene [chr2:179462295 NM_133378(TTN); exon243: c.49810A>T (p.R16604X)] (**Figure 1**).

Digoxin, captopril, and hydrochlorothiazide were given to manage heart failure. The digoxin concentration ranged between 0.15 and 0.22 µg/l. Dosage and adjustment are shown in **Table 1**.

**Table 1:** Medications for heart failure.

Timeline	Medication	Dosing
2021.9.15-2021.10.31 (home medication)	Digoxin	6 µg/kg/day, po
2021.11.1-now	Digoxin	62.5 µg, q12h, po Hold a dose if HR <90 bp
2021.11.1-now	Hydrochlorothiazide	12.5 mg, q12h, po
2021.11.1-now	Spironolactone	10 mg, q12h, po
2021.11.1-now	Coenzyme Q10	10 mg, bid, po
2021.11.1-now	Captopril	4.17 mg, q12h, po Hold a dose if BP <90/60 mmHg

**Figure 2:**
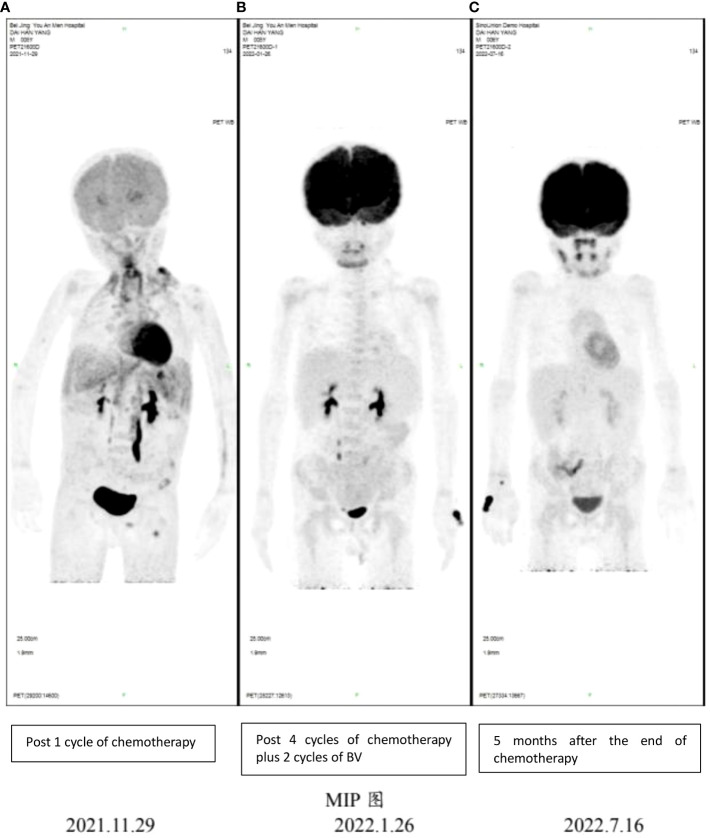
PET-CT imaging showing early response following 4 cycles of chemotherapy plus 2 cycles of BV and sustained remission in this patient. **(A)** PET-CT results for the patient on 11/29/2021; **(B)** PET-CT results for the patient on 01/26/2022; **(C)** PET-CT results for the patient on 07/16/2022.

Referring to the CCG trial C5942, group 3 chemotherapy was planned to the patient (8). Considering the poor cardiac function, Adriamycin AVPC (CTX 1.2 g/m^2^, d1–2; VCR 1.4 mg/m^2^, d1 (max 2 mg); MP 250 mg/m^2^, d1; Pred 60 mg/m^2^, po, d2–4) was removed. However, the desired treatment effects were not reached (see **Figure 2A**). EC (Ara-C 3 g/m^2^. d, d1–2; VP-16 200 mg/m^2^, d1–2) then followed. BV was added to the plan starting from the third cycle of chemotherapy. The treatment plan, specific dose, and adjustment are shown in **Table 2**.

**Table 2:** Chemotherapy schedule and dosage.

Cycle	Hospital stays	Plan	Composite
Cycle 1	17 days	AVPC(-ADR)	CTX 1.2 g/m^2^, d1-2; VCR 1.4 mg/m^2^, d1(max 2 mg); MP 250 mg/m^2^, d1; Pred 60 mg/m^2^, po, d2-4
Cycle 2	5 days	EC	Ara-C 3 g/m^2^.d, Q12h, d1-2; VP-16 200 mg/m^2^, d1-2
Cycle 3	4 days	BV+COPP/ABV (-ADR)	BV 1.8 mg/kg, d1; CTX600 mg/m^2^, d1; VCR 1.4 mg/m^2^, d1(max 2mg); PCZ100 mg/m^2^, po, d1-7; Pred 40 mg/m^2^, po, d1-14; BLM 10 U/m^2^, d8; VLB 6 mg/m^2^, d8
Cycle 4	5 days	BV + AVPC(-ADR)	BV 1.8 mg/kg, d1; CTX 1.2 g/m^2^, d1-2; VCR 1.4 mg/m^2^, d1(max 2 mg); MP 250 mg/m^2^, d1; Pred 60 mg/m^2^, po, d2-4
Cycle 5	3 days	BV+EC	BV 1.8 mg/kg, d1; Ara-C 3 g/m^2^.d, Q12h, d1-2; VP-16 200 mg/m^2^, d1-2

CTX, cyclophosphamide; VCR, vincristine; ADR, Adriamycin; MP, methylprednisolone; Pred, prednisone; Ara-C, cytosine arabinoside; VP-16, etoposide; BV, brentuximab vedotin; PCZ, methylhydrazine; BLM, bleomycin; VLB, vinblastine; -ADR, remove ADR.

**Figure 3:**
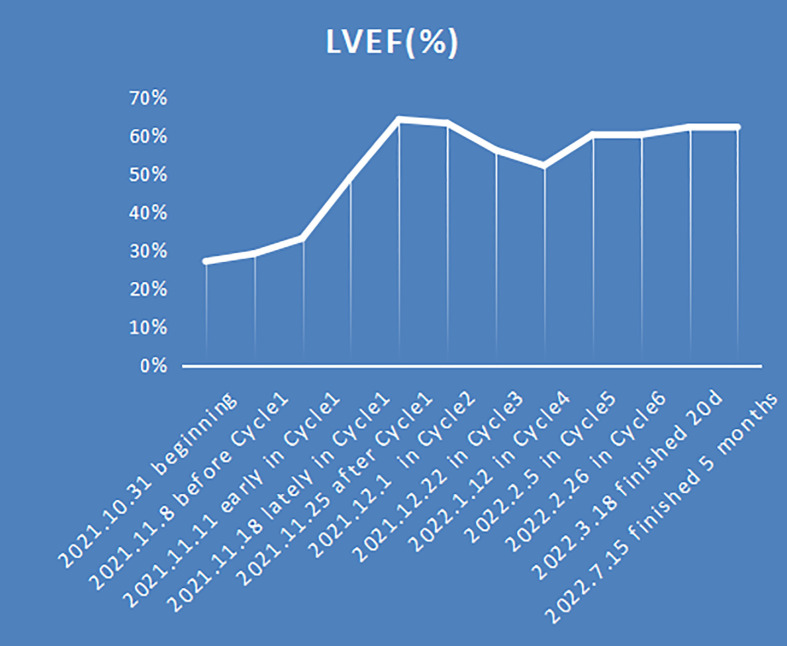
LVEF (%) changes with treatment.

PET-CT scan was not performed initially because of the patient’s condition and high acuity status. However, PET-CT still showed systemic tumor invasion after the first course of chemotherapy (**Figure 2A**). After the fourth cycle of chemotherapy (2 cycles plus BV), the tumor was reduced by over 85% compared with the initial stage of the disease, and the effect was evaluated as complete remission (CR) according to Lugano 2014 Criterion (9) (**Figure 2B**). At the end of all six cycles of chemotherapy, the tumor state was still CR. No rash, fever, cough, dyspnea, nausea, or vomiting was reported. Grade 2–3 hematological toxicity occurred after combined chemotherapy, mainly manifested as neutropenia without infection. No neuropathy such as burning sensation and neuropathic pain and no pulmonary toxicity were observed. During the 11-month follow-up, the child was still in complete remission (**Figure 2C**), and LVEF had recovered to 62% (**Figure 3**). There was no abnormality in thyroid function, electrolytes, liver, and kidney function.

## Discussion

This case report firstly reports the usage of BV in pediatric HL patients with TTN gene mutation. Due to the lack of best practice evidence, the possibility of cardiotoxicity from anthracycline-containing chemotherapy in patients with TNN gene mutations (10), and the fact that lack of anthracycline-containing chemotherapy as backbone may result in suboptimal tumor response (11), BV was substituted for doxorubicin to treat this patient. As a result, the patient achieved complete remission with no significant adverse events.

TTN gene mutation is associated with dilated cardiomyopathy (12). This patient had TTN truncating mutations, which can interrupt the production of titin protein, resulting in decreased myocardial cell elasticity, manifested by bilateral ventricular dilatation and decreased left ventricular function. At the same time, the child was thin with moderate anemia, experiencing cachexia, which increased the burden on the heart. Under the simultaneous action of internal and external factors, his cardiac function was initially extremely poor.

Linschoten et al. (4) reported that two female breast cancer patients with TTN gene mutation experienced decreased myocardial contractility and significantly decreased LVEF after chemotherapy containing ADR. In addition, Garcia-Pavia et al. (3) showed that TTN truncating variants are a susceptibility factor for cardiomyopathy after chemotherapy in adult and childhood cancer patients. Therefore, we withdrew ADR from the original plan to avoid potential cardiovascular damage in this patient.

However, chemotherapy without ADR has a decreased antitumor effect, as the first PET-CT scan (29 November 2021) showed systemic tumor invasion. Therefore, BV shed light on the treatment for this patient. Some researchers have tried to use BV combined with chemotherapy as the first-line treatment for children with HL and even BV monotherapy. These attempts have shown promising results (10). Metzger et al. (6) replaced vincristine (VCR) with BV in the first-line treatment of children with high-risk Hodgkin lymphoma. The results showed that the children were highly tolerable to BV combined with chemotherapy, and this attempt can avoid the neurotoxicity caused by VCR and produce an excellent therapeutic effect.

However, combining BV with bleomycin (BLM) may lead to an increased risk of pulmonary toxicity (11). Considering that if both ADR and BLM are removed, the antitumor effect may be further reduced, and the disease may progress to a refractory HL. Therefore, we allowed the combination of BV and BLM for two cycles of treatment and monitored for pulmonary toxicity. Choi et al. (5) studied the use of BV instead of BLM for the first-line treatment of adult patients with stage III/IV Hodgkin lymphoma. The results showed a reduced incidence of pulmonary toxicity and high progression-free survival and overall survival.

## Conclusion

The combination of chemotherapy (no ADR) with BV is effective and well-tolerated for the treatment of HL pediatric patient with dilated cardiomyopathy. It should be considered as first-line used to achieve rapid tumor remission.

## Data availability statement

The original contributions presented in the study are included in the article/supplementary material, further inquiries can be directed to the corresponding author.

## Ethics statement

Written informed consent was obtained from the individual(s), and minor(s)’ legal guardian/next of kin, for the publication of any potentially identifiable images or data included in this article.

## Author contributions

YL and LL did the acquisition, analysis, and interpretation of data. HS, NL, and SH participated in the treatment of the patient and revised the manuscript. AO, XX, and XW revised the manuscript. YD contributed to the conception, drafted the work, participated in the interpretation, and revised the manuscript. All authors read and approved the final manuscript.

## Conflict of interest

The authors declare that the research was conducted in the absence of any commercial or financial relationships that could be construed as a potential conflict of interest.

## Publisher’s note

All claims expressed in this article are solely those of the authors and do not necessarily represent those of their affiliated organizations, or those of the publisher, the editors and the reviewers. Any product that may be evaluated in this article, or claim that may be made by its manufacturer, is not guaranteed or endorsed by the publisher.

## Abbreviations

HL, Hodgkin’s lymphoma; LDCHL, lymphocyte depleted classic Hodgkin lymphoma; IHC, immunohistochemical staining; COVID-19, Corona Virus Disease 2019; WES, whole exome sequencing; NGS, next-generation sequencing; CT, computerized tomography; PET-CT, positron emission tomography-computerized tomography; BSA, body surface area; CTX, cyclophosphamide; VCR, vincristine; ADR, Adriamycin; MP, methylprednisolone; Pred, prednisone; Ara-C, cytosine arabinoside; VP-16, etoposide; BV, brentuximab vedotin; PCZ, methylhydrazine; BLM, bleomycin; VLB, vinblastine; TTN, titin; CR, complete remission; CHIPS, Childhood Hodgkin International Prognostic Score.
